# Assessment of older persons with multimorbidity in Norwegian primary care: a qualitative study of healthcare professionals’ experiences and preferences in fostering continuity of care

**DOI:** 10.1186/s12913-024-12185-4

**Published:** 2025-01-02

**Authors:** Turid Rimereit Aarønes, Kristin Taraldsen, Linda Aimée Hartford Kvæl

**Affiliations:** 1 Faculty of Health Sciences, Department of Rehabilitation Science and Health Technology, OsloMet – Oslo Metropolitan University, Oslo, Norway; 2 Department for Research, Innovation, Education, and Health Service Development, Møre og Romsdal Hospital Trust, Ålesund, Norway; 3 NOVA – Norwegian Social Research Institute, Department for Ageing Research and Housing Studies, OsloMet – Oslo Metropolitan University, Oslo, Norway

**Keywords:** Assessment, Interdisciplinary collaboration, Care continuity, Person-centred care, Holistic understanding, Primary care, Professional communication, Multimorbidity

## Abstract

**Background:**

As the population ages, more people live longer with multimorbidity. Older people with multimorbidity face diverse needs and medical conditions, increasing the risk of adverse health outcomes, and often experience fragmented healthcare. Research has called for better ways to reach, understand and care for this group to enhance care continuity. This study aimed to examine healthcare professionals’ experiences and preferences as they relate to assessments’ role in promoting care continuity for home-dwelling older patients with multimorbidity in community-based healthcare.

**Methods:**

This qualitative study acquired qualitative data from 17 healthcare professionals from reablement teams, interdisciplinary teams, rehabilitation teams and home nursing in three Norwegian municipalities. Representing nursing, physiotherapy, occupational therapy and social work, all participants were experienced in assessing older home-dwelling patients with multimorbidity. Semi-structured focus group and individual interviews were conducted, then the interviews were transcribed and analysed using reflexive thematic analysis.

**Results:**

The analysis elicited three themes: gaining insight beyond diagnoses to promote relational continuity, facilitating interaction to ensure informational continuity, and linking patient journeys to facilitate managerial continuity. The themes underscore the significance of evaluating patients beyond their medical conditions, emphasising assessment’s collaborative nature across disciplines. Healthcare professionals use diverse assessment methods and facilitate interaction to understand patients’ needs. Working together across different healthcare professions is key for care that includes the whole patient, but challenges such as underutilisation of assessments and poor documentation still exist. Furthermore, linking patient journeys remains difficult due to fragmented services and limited resources. Despite these challenges, assessments were viewed as crucial to care continuity.

**Conclusions:**

In this qualitative study, healthcare professionals emphasised that assessment is a complex, continuous process due to the fluctuating health of individuals with multimorbidity. Effective instruments and diverse assessment methods are essential to understanding all aspects of patients’ health and well-being to ensure care continuity across individual, service, and system levels. Our findings highlight the need for systematic and structured use of assessments to improve interdisciplinary collaboration and personalised care for older individuals with multimorbidity. Understanding the patient journey is crucial for achieving these goals, potentially benefiting healthcare professionals, policymakers, and primary care providers.

**Supplementary Information:**

The online version contains supplementary material available at 10.1186/s12913-024-12185-4.

## Background

In Norway and other Western nations, helping older adults continue living at home as they age aims to preserve their independence and community connections while managing the aging population’s socioeconomic impact [1, 2]. As more and more older individuals aim to keep living at home, addressing their self-care needs is essential [3]. The global population has been aging, as more people live longer, but with functional limitations and multimorbidity, which is defined as having two or more chronic conditions [4, 5]. Multimorbidity becomes more common with age [6–8], affecting over 50% of those age 60 and up in community settings [9]. Shorter hospital stays, reduced institutional capacity, and rising demand for health services have heightened home-based care’s complexity [1, 10]. Older home-dwelling adults with multimorbidity face varied needs [5, 11], increasing their risk of adverse health outcomes, such as functional decline, treatment complications, reduced quality of life, depression, and frailty [5, 8, 12]. Furthermore, managing multiple conditions, medications, and interactions with healthcare professionals (HCPs) across services and care levels [5] leads to higher healthcare utilisation [13].

With several different HCPs often used to treat each in-home patient, effective assessments that promote interdisciplinary and HCP-patient collaboration are needed. Despite guidelines recommending holistic and seamless services, older adults with multimorbidity experience fragmented care [14, 15]. To counteract fragmented services, the World Health Organisation recommends implementing more coordinated and person-centred primary care [4], and has introduced the Integrated Care for Older People (ICOPE) framework [16]. ICOPE includes screening, assessment and management of intrinsic capacity declines (i.e., a patient’s combined physical and mental abilities) [16]. Based on this, we view assessment as an ongoing process that incorporates physical, psychological and social aspects of patients’ health. Assessment is particularly crucial in caring for older home-dwelling patients with multimorbidity, as it helps identify individual needs and ensures appropriate care. Multimorbidity can lead to frailty, resulting in increased vulnerability and adverse health outcomes; thus routine assessments for frailty is recommended for individuals with multimorbidity [14].

Municipal healthcare represents the lowest level of formal care in the Norwegian healthcare system, delivering services to all residents, including home-dwelling older patients [17]. A comprehensive approach to patient care for older individuals with multimorbidity is needed as responsibilities shift from specialised to primary care [11, 18]. With patient care’s growing complexity, HCPs in primary care need to navigate the complex interplay between practical, social and psychological factors [15, 19–21]. A holistic approach includes considering patient preferences, values and voices, emphasising bio-psycho-social care models, rather than focusing only on physical health conditions [22]. While integrated care promotes seamless transitions between providers, person-centred care places the person at the centre, aiming to ensure a meaningful life through coordinated support tailored to individual needs [5, 23, 24]. Therefore, managing multimorbidity in municipal healthcare requires that HCPs have expertise and skills in various forms of assessment [5].

Assessment involves gathering and analysing information to inform judgements [14]. Understanding how HCPs manage care for older patients with multimorbidity is essential, by considering interactions between health conditions and treatments as they relate to patients’ quality of life [14]. Understanding individuals’ health status helps HCPs tailor interventions effectively, improving condition management and care continuity across settings [25]. Municipal healthcare continuity entails offering consistent, personalised care over time while considering patients’ values, relationships, and care plans. Continuity encompasses relational, informational, and management dimensions [26]. Relational continuity is nurtured through ongoing therapeutic relationships and consistent interactions between HCPs and patients. Involving patients and their families in goal setting fosters interprofessional collaboration and links assessment to the relational aspect of care continuity [27]. Furthermore, HCPs maintain informational continuity through assessment by personalising healthcare and coordinating care across providers [26]. Finally, municipal healthcare promotes management continuity through ongoing assessments of patients’ evolving needs and care transitions.

As noted earlier, older patients with multimorbidity should receive routine frailty assessments to evaluate physical, psychological and social functioning while incorporating various health and lifestyle risk factors [28, 29]. However, choosing the right frailty instrument is complex due to the absence of standardisation [29], the numerous options available, and the need for more consensus on their usage [30]. Assessments within municipal healthcare are challenging and often limited by short visits from different HCPs [5]. Challenges include fragmented care [6, 12], which can lead to duplication or gaps [31], and limited resources, which hinder the multidisciplinary care needed for comprehensive assessments. Examining HCPs’ experiences with assessment is crucial to understanding how they navigate obstacles [32], particularly how limited resources impact assessments’ thoroughness and effectiveness in municipal healthcare for this population [33].

Caring for older home-dwelling patients with multimorbidity is a complex task. Existing literature has revealed gaps in our comprehension of how HCPs conduct assessments to identify individual care needs in managing multimorbidity and frailty [19, 34]. We need to better understand how HCPs use the information they gather from assessments in their practices [35]. We also need more insights into how a person’s health problems and personal life situations influence HCPs’ assessments [32, 36]. As older patients themselves have emphasised, interprofessional communication and assessment are important in ensuring care continuity [37]. How HCPs facilitate continuity within multidisciplinary teams needs examination [32], including how HCPs collaborate and communicate on assessments [38, 39]. Understanding the role of assessments of older patients with multimorbidity and mitigating barriers to seamless transitions between care levels are essential in facilitating care continuity [20, 26]. Understanding how assessments are conducted to facilitate continuity in multimorbidity management can help tailor care to each person’s individual needs and circumstances [40]. Thus, this study aims to examine HCPs’ experiences and preferences as they relate to assessments’ role in promoting care continuity for home-dwelling older patients with multimorbidity in community-based healthcare. This study can help guide development of more efficient ways to provide healthcare and improve the care experience for older patients living with multimorbidity.

## Methods

### Design

This study adopted a qualitative pragmatic approach, employing both focus group (FG) and individual interviews with HCPs. A pragmatic position involves viewing knowledge as situated and balancing theoretical rigor with practical considerations to elicit findings that are valuable and applicable in real-world contexts [41].

### Context: community healthcare services

The public healthcare system in Norway comprises specialist and primary care levels. Four regional health authorities manage specialist care in hospitals, while municipalities are responsible for primary care and social services [18]. Specialist and primary healthcare services collaborate closely to provide appropriate, timely care at the lowest level [42]. Early discharge from hospitals requires efficient assessments and seamless coordination between specialist and primary care, not only during transition periods, but also for continued treatment, to meet patients’ needs adequately [17]. Municipal services are delivered based on individual needs, ranging from basic home care and home nursing to institutional stays. Rehabilitation services, which older patients with multimorbidity utilise frequently, are offered through short-term stays in nursing homes or reablement services [43]. We conducted the study within municipal healthcare services in one medium-sized and two large municipalities in Central Norway. Our participants worked in various healthcare settings, including home care, round-the-clock nursing and medication management services. The settings also included an institution-based rehabilitation ward comprising 14 beds managed by a multidisciplinary team. Furthermore, reablement teams and municipal physio- and occupational therapy offering home-based multidisciplinary assessments and rehabilitation aimed at restoring and improving functions and independence were included. All settings were relevant for older patients with multimorbidity. Their varying functional levels, coupled with a desire to get them home and for them to remain at home for as long as possible, highlight the need for solid assessments and coordinated municipal services at different stages [5].

### Sample and recruitment

We recruited HCPs who had direct experience managing older patients with multimorbidity. Our sample was strategic [44], using these inclusion criteria: HCPs working with individuals age 65 years and up in home care services or interdisciplinary teams with at least one year of employment and currently in a 50% position or higher. We aimed to form a diverse group to capture various perspectives on these services, reaching out to leaders within relevant municipalities’ health services to connect with eligible HCPs. Initially, we sent information through email. Following this, one leader wanted to meet with us, while the others received information via phone. Subsequently, the HCPs were invited to participate either through their leaders or by responding directly to the researcher. HCPs interested in participating signed an informed consent form before the interviews took place. The number of participants depended on the richness of the information provided and was adjusted based on the information density obtained by consensus through the author team [45]. We decided to conduct both FG and individual interviews with a selection of participants that represented our specific context realistically.

### Data collection

We conducted seven interviews between January and September 2023, including two FGs and five individual interviews. The two FG interviews involved eight and five participants. As one FG was larger, we needed to go into more depth for elaboration and information density and decided to conduct a follow-up interview. Therefore, one individual interview served as a follow-up after the first FG session. The first author led the FG interviews, with the last author serving as an observer, and the first author led the individual interviews alone. The FG interviews lasted between 61 and 75 minutes each, and the individual interviews lasted between 55 and 74 minutes each. All interviews were recorded as MP3 files.

The FG interview guide was developed initially followed by an almost identical individual interview guide, as practical decisions were made to conduct both FG and individual interviews. Thus, two interview guides were tailored to accommodate FG and individual interviews, ensuring a thorough examination of the participants’ experiences and preferences regarding care of older patients with multimorbidity (see interview guides in Additional files 1 and 2). Developing our own interview guides was part of our pragmatic position to examine HCPs’ experiences and preferences without being confined to existing literature. The topics included descriptions of various assessment methods, participants’ experiences with these methods, integrating patient input during assessments and identifying barriers to and facilitators of assessment. Developed collaboratively by the author group and informed by consultation with a reference group comprising HCPs and a user representative, the interview guides aimed to capture valuable insights from diverse perspectives. Throughout the interviews, the interview guides helped maintain conversational flow and examine both overarching themes and important subthemes. Following the initial individual interview, adjustments were made to the interview guides to prioritise open-ended questioning to ensure that participants could share their perspectives freely. The facilitator guided the interviews using a responsive interviewing style [46] to ensure that relevant topics were addressed while placing participants’ descriptions, experiences, and thoughts at the forefront of the conversation.

### Data analysis

The first author transcribed the interviews verbatim and analysed them using the software tool HyperResearch. Following Braun and Clarke [47], an experiential-oriented reflexive thematic analysis was employed. All authors independently reviewed the transcripts and convened to discuss and examine the core findings. The first and last authors then independently coded all transcripts closely aligned with the material. The codes were organised into initial themes to capture the data’s essence. Table 1 illustrates how quotes were given code labels and subsequently categorised into initial themes.

**Table 1:** The process from quote to code label and initial theme

**Quote**	**Code label**	**Initial theme**
‘However, you can observe that individuals with multimorbidity are more exposed to disruptions in their care plans. For example, a urinary tract infection or fall can quickly make them unable to continue as before’ (HCP 7, physiotherapist)	The system can be disrupted easily in multimorbid patients	A Complex Service

Altogether, 12 initial themes were identified, then thoroughly discussed among the author team and refined further through iterative dataset analysis. We found continuity to be the overarching concept connecting the initial themes; thus, we utilised the care continuity dimension [26] as a framework for the analysis. We then established and discussed the three main themes based on different aspects of continuity on individual, service and system levels.

The authors have broad clinical and research experience relevant to the study’s topic. This expertise might enrich our interpretation of the data, as well as reveal potential preconceptions.

## Results

### Participants

Altogether, 17 participants were included – comprising nurses (*n* = 9), physiotherapists (*n* = 5), occupational therapists (*n* = 2) and a social worker (*n* = 1) – with an average age of 38. Eight participants worked in home care services (home nursing, occupational and physiotherapy services) and nine were part of interdisciplinary teams (reablement teams, ambulatory rehabilitation teams and institutional rehabilitation teams). The participants had worked in their current settings for an average of eight years, and eight held additional qualifications in healthcare, rehabilitation and social sciences, including two with master’s degrees. Three participants worked as team leaders, one as an assistant leader and coordinator, one as a specialised nurse, three as specialist physiotherapists, and one as a specialist occupational therapist. The study participants’ background characteristics are presented in Table 2.

**Table 2:** Study participants’ background characteristics (*n* = 17)

**Characteristics**	***n*=17**
**Gender**	
Female	17
**Age in years**	
<30	4
30 – 39	5
40 – 49	7
50 – 59	1
**Years of experience in the current workplace**	
<5	6
5 – 10	7
11 – 15	2
16 – 20	1
21 – 25	1
**Municipality**	
A	8
B	4
C	5

### Main themes

The HCPs described assessing home-dwelling individuals with multimorbidity as a multifaceted process within a complex healthcare setting. They emphasised that assessment extends beyond mere testing; it demands skills, clinical expertise and ethical considerations. The HCPs used various assessment methods and stressed the importance of both structured and unstructured approaches to gather information. They found assessment crucial to ensuring care continuity, leading to our development of three main themes: gaining insight beyond diagnoses to promote relational continuity, facilitating interaction to ensure informational continuity and linking patient journeys to facilitate managerial continuity (outlined in Table 3 below).

**Table 3:** Themes

**Initial themes**	**Sub themes**	**Main themes**
The vulnerable patient	Unlocking trust: the conversation connection	Gaining insight beyond diagnoses to promote relational continuity
Positioning for trust		
A holistic approach	Examining the patient perspective	
Interaction and coherence		
Assessment’s purpose and meaning	Assessment demands expertise and discretion	Facilitating interaction to ensure informational continuity
Interdisciplinary collaboration		
The clinical lens on assessment	Integrating written and oral information	
Documentation: binding care together		
Relatives caught between different opinions	Understanding the patient journey	Linking patient journeys to facilitate managerial continuity
Change and challenge when ‘everyone’ is going home		
Will, but not resources	Limited flexibility in a standardised system	
A complex service		

### Gaining insight beyond diagnoses to promote relational continuity

The HCPs emphasised the importance of recognising the individual beyond diagnoses, with an emphasis on understanding patients’ values and skilfully guiding assessment conversations.

The participants noted that assessment conversations went beyond standardised forms or instruments and were framed to cultivate trust and security in a safe and relaxed atmosphere, an aspect viewed important for positioning oneself for an optimal assessment. As one participant noted:

*‘I see this assessment tool more as a work tool or a to-do list, rather than something to strictly adhere to. The real value lies in having a meaningful conversation with the patient, where you naturally delve into their concerns. You gain much more insight from the patient through such conversations compared to just sitting with the assessment tool in hand. Writing notes might create a different experience for the patient during the visit, I believe’* (HCP12, occupational therapist).

The HCPs viewed establishing a trusting relationship as crucial to gathering thorough information during the first assessment through an atmosphere of openness and mutual understanding. They conducted assessments that encompassed physical, psychological and social aspects alongside clinical insights. As one participant noted:

*‘I believe that if patients are genuinely honest during the initial assessment, it provides a thorough understanding. We discuss their social connections, nutrition, mental health, and substance use, covering various topics. We really get to know them well since we have a close relationship, and there are only four of us, so they usually become familiar with us. They tend to open about a lot during the assessment’* (HCP10, physiotherapist).

Several HCPs emphasised the significance of observing and assessing patients over time in different contexts to evaluate their functional levels accurately. This approach helped the HCPs understand how patients adapted, with one physiotherapist noting its importance in preventing unnecessary provision of aids, as patients may adjust to their circumstances over time:

*‘Before introducing too many aids [in the patient’s home], it’s important to observe how they adapt, ensuring we don’t inadvertently hinder their mobility or take away something they’re on the verge of regaining’* (HCP11, physiotherapist).

In emphasising the importance of thoroughly examining patients’ perspectives, the HCPs highlighted the significance of interaction and coherence in their care practices to establish common patient goals. For example, one participant described how the reablement team collaborated with other HCPs during weekly meetings to identify patients who could benefit from interdisciplinary approaches. The HCPs gained valuable insights by asking patients ‘What matters to you?’ However, the HCPs also described challenges in establishing good relationships to elicit the patients’ perspectives, particularly when time and expertise were limited, hindering creation of a safe and open environment. The HCPs were concerned that poor communication and resource constraints led to inadequate information exchange among staff and poor patient follow-up. This lack of interprofessional collaboration was identified as a barrier to comprehensive patient knowledge, with one physiotherapist highlighting the importance of asking what matters to patients:

*‘We sometimes receive referrals where the home service believes the patient should manage support stockings independently. However, upon assessment, we realise that’s not the priority. What truly matters is their ability to climb stairs to reach the daycare centre, socialise with friends, or whatever else is important to them. It’s a fantastic way to learn more about their priorities’* (HCP10, physiotherapist).

The participants relied on their clinical judgement during assessments, recognising its importance in maintaining patient rapport and care continuity. They emphasised that assessments went beyond documented information, with familiarity enabling them to detect changes through observation, highlighting assessment’s ongoing nature. As one participant noted:

*‘When we visit patients at their homes, we always assess them. We’re especially observant of those with multiple diagnoses who we know are frailer. As we get to know them, we work with them differently, relying heavily on our clinical judgement’* (HCP2, nurse).

To summarise, the theme entails assessment’s critical role in establishing enduring bonds between HCPs and patients, emphasising the importance of viewing individuals beyond their medical diagnoses and recognising assessment’s collaborative nature across disciplines.

### Facilitating interaction to ensure informational continuity

The HCPs used assessments to share patient information and ensure service consistency through collaboration both within internal teams and with external partners.

The participants stressed the importance of using structured assessment methods, such as various instruments, and unstructured methods, such as observation and informal conversations. They highlighted the significance of expertise and professional judgement in conducting these assessments to describe patients’ conditions comprehensively.

Many participants described how assessment instruments functioned as checklists, particularly during patient transitions, to identify areas in which patients were independent or needed further assistance. The HCPs expressed that assessments served as valuable tools during meetings among different HCPs, fostering collective discussions of patients during transitions. This collaborative process relied on a shared understanding among team members regarding the assessment instrument and its interpretation. As one participant noted:

*‘It is a useful document for interdisciplinary meetings. It helps us cover all necessary points before patients go home and serves as a good checklist for those managing their rehabilitation and discharge. These are essential things to know before a patient can go home. Being able to review and start training on these items as soon as they arrive is valuable, but the technical aspect of the document still needs improvement’* (HCP1, nurse).

The participants emphasised interdisciplinary collaboration as being essential for gaining a comprehensive understanding of patients, particularly those discharged early from the hospital who often face complex challenges beyond physical needs. Different professional groups observed unique patient aspects and communicated diversely, contributing to holistic views and new insights. As one nurse noted:

*‘No, because then it becomes more interdisciplinary, which is beneficial, and it becomes more intensive. You realise that when there are three of us, we can provide follow-up, almost every day, each with different perspectives, allowing us to address the whole person comprehensively’* (HCP9, nurse).

Many participants were concerned about underutilisation of assessments and a lack of recognition of assessments’ value, leading to frustration and a perception that assessments could lose their intended purpose through diminished effectiveness and impact. The participants stressed the importance of assessing all patients and fostering a shared approach across services to reduce inconsistencies in measures and interventions. As one occupational therapist noted:

*‘We need to agree on the same thing, like Mr. Olsen going shopping on Tuesdays. It’s a mutual goal, ensuring he gets help earlier that day and doesn’t have his mail collected every day as he needs to practice walking that distance. So, having a common understanding in assessments is crucial. There should be a shared overall goal in the journal system. What are we working towards? But it’s not working optimally…’* (HCP12, occupational therapist).

Our participants described how inconsistencies in practices came from inadequate training and using multiple assessment instruments across different organisational units and levels, eliciting confusion about assessment objectives and preventing effective communication. Each municipality developed its own assessment form because they felt the need to integrate each profession’s assessments into a unified interdisciplinary format to prevent work duplication and repetitive patient questioning. As one participant noted:

*‘Yes, the ADL-checklist created by a working group here delves deeper and may have a more relevant focus area for us compared to other instruments, especially for those familiar with the service ‘ […] ‘That’s how we can describe the patients in that form because it allows us to provide an explanation: ‘Yes, they can, but …’ [followed by why the patient doesn’t do something despite being able to]’* (HCP3, social worker)

The participants stressed the importance of documentation, calling it the glue that hold care together. Several said that a thorough assessment was only useful if it was well-documented. A comprehensive assessment of all patients was viewed as crucial in identifying and implementing appropriate measures, and facilitating patients’ return home. Furthermore, fragmented services were observed due to disconnected journal systems, and assessments were underutilised during patient transfers. One home nurse commented on the issue of different journal systems:

*‘You can quickly fall between two stools because they have to see the GP. That’s one thing, but they may have to visit three different outpatient clinics as well … and then there are 10 different journal systems which don’t communicate …’* (HCP5, nurse).

To summarise, this theme underscores how HCPs facilitate interaction and use varied assessments for comprehensive insights, emphasising interdisciplinary collaboration to gain a holistic understanding of physical, emotional, social, and environmental needs. Challenges include underutilised assessments and disconnected documentation systems.

### Linking patient journeys to facilitate managerial continuity

The HCPs explained that they found the patient journey complex and described challenges in navigating a system in which they felt they had limited influence. The participants described a patient group that typically interacts with numerous professionals across different care levels.

Some HCPs suggested standardising assessment instruments across municipal and hospital settings to enhance care continuity. They noted that the patient journey became disjointed when information was transferred inconsistently between HCPs. Many participants said patients and their relatives often felt stranded due to insufficient information and communication gaps between services. Moreover, assessment inconsistencies, compounded by using various assessment instruments, contributed to fragmented services. As one nurse noted:

*‘Yes, I often see that with those who are very ill… they frequently have changes in their medications… There could be a lot of interactions… when the GP prescribes one medicine and the nephrologist prescribes another, and then they’re supposed to communicate, but we don’t really feel like they do because there’s a lot of back and forth… It becomes a trial project…’* (HCP5, nurse).

Some HCPs expressed concerns about the inefficiency of conducting comprehensive assessments for patients with limited rehabilitation potential, citing time constraints as a significant factor. They felt that a shortage of short-term nursing home spaces prioritised quick discharge over thorough assessments, leading to insufficient information and unnecessary readmissions. Furthermore, poor communication between services exacerbated challenges in directing patients to suitable care. Lack of assessments made it difficult to identify individuals needing interdisciplinary services in the municipality. Consequently, the HCPs wanted to see patients earlier to address these challenges effectively. As one nurse stated:

*‘So … we struggle a bit to catch those who need that interdisciplinary approach … I think, in our municipality, and it has basically lasted. And it goes up and down a bit. And the lack of precision when it comes to correct use of our institutional places. Because lately we have had a lot of people who are very ill, who have been granted a rehabilitation stay only to be admitted to the hospital the next day, and that’s not rehabilitation’* (HCP9, nurse).

In addressing complex patient care, the HCPs focussed on coordinating services rather than engaging directly with patients. Moreover, inpatient rehabilitation HCPs highlighted resource scarcity for patients transitioning from short-term stays to returning home. One participant underscored the critical need for care continuity:

*‘Because there is so much going on everywhere … so many could benefit from a coordinator … very few have that one person with a full overview … and that’s often necessary. At every level of care, there are crucial aspects to address and follow up on, compounded by involvement from various professionals. There are a lot of cooks, and a lot of good is being done that might go unnoticed due to communication gaps. It’s very challenging. You can’t document everything either, because then you won’t have time for the patients’ (HCP 13, physiotherapist)*

All participants agreed on holistic care’s importance, but simultaneously expressed concerns about lacking time and resources for its proper implementation. According to them, feeling rushed led to insecurity, discontinuity in services and duplicated efforts. One participant highlighted the repercussions of limited time and resources on HCPs’ ability to capture a holistic view and provide comprehensive services:

*‘Patients feel a lack of coherence in the service, leading to a perception that HCPs do not collaborate effectively. This arises when different professionals convey conflicting information or duplicate efforts, resulting in the inefficient use of resources’* (HCP12, occupational therapist)

Many HCPs expressed frustration with their limited ability to exercise professional judgement within a standardised system, hindering thorough assessments and personalised care. Furthermore, budget cuts had ended preventive efforts, resulting in short-term stays being offered mainly post-hospitalisation, rather than proactively to home residents. This often meant that patients who were too frail to go home were sometimes discharged early because short-term stay beds were filled with patients needing long-term care. One nurse highlighted this practice’s consequences:

*‘Or when someone is actually frailer or… they’re going home, or they have to go home, but we’re hesitant to send them home, yet… they’re expected to manage at home, and then after two days, they end up in the immediate help department at the emergency room’* (HCP15, nurse)

To summarise, the theme reveals challenges in linking patient journeys due to inconsistent assessments, poor communication between services, and limited resources. Despite these challenges, the participants highlighted assessments’ important role in improving managerial continuity, fostering better communication, consistency and coordinated care across the patient journey.

## Discussion

This qualitative study examined HCPs’ experiences and preferences regarding assessment of older patients with multimorbidity in municipal healthcare. The main findings indicate that assessment is a multifaceted and continuous process conducted within a challenging context. The results highlight the importance of building strong HCP-patient relationships for comprehensive and holistic evaluations. Assessments can serve as boundary objects, helping HCPs interact effectively within teams and with external partners. Furthermore, systematic assessments foster care coordination and connect patient journeys, despite challenges such as multiple HCPs involved, disconnected journal systems, limited resources, and underutilised assessments.

We found that the HCPs used structured and unstructured methods to assess various aspects of patients’ health and well-being. This approach is crucial because multimorbidity impacts all aspects of a person’s life – including their environment, relationships, and health – leading to diverse and unique health issues that guidelines often cannot fully address [11, 32]. Therefore, our findings align with other studies in emphasising the need for a multifaceted approach to understand and address patients’ multimorbidity needs comprehensively [21, 25, 48]. Multimorbidity’s complexity includes coordination and communication challenges [49]. Our findings align with this, demonstrating that assessments are complex processes due to the need to consider multiple health dimensions, various HCPs’ involvements and limited time. The HCPs highlighted the preference for using a multidimensional assessment approach to understand all aspects of patients’ health and well-being, and to get to know the people behind diagnoses. Other studies also emphasised the need for a holistic approach to meet patients’ needs [5, 14, 40, 50]. Assessing multiple dimensions integrates well with the bio-psycho-social model of health, acknowledging that multiple aspects contribute to health outcomes [21, 51, 52]. Thus, assessments are essential in identifying individuals at risk of frailty or those who are already frail and need personalised interventions to prevent further functional decline and adverse outcomes [53, 54].

Attentive communication is crucial for person-centred care, particularly when caring for older people [55]. Our HCPs emphasised the importance of framing assessment conversations to build mutual trust and understanding with vulnerable patients, aligning with studies that underscore strong patient relationships’ essential role in meeting individual needs [21, 50, 56]. Therefore, we interpreted that the guiding principle of individualised treatment in multimorbidity care [5] highlights the potential from thorough assessments. The HCPs gained trust through communication and getting to know patients, thereby establishing therapeutic relationships. Previous research emphasises communication skills’ importance and, thus, how assessments promote quality care and patients’ confidence in HCPs’ assessments [54, 55].

Several studies underscore the importance of examining what matters to patients, symbolising person-centred care [5, 32, 57, 58], and the HCPs in our study asked their patients the question ‘What matters to you?’ as part of their assessments. Previous research [59] found that asking ‘What matters to you?’ to be a complex process that needs a competent framing in clinical practice, in accordance with our HCPs who experienced how cultivating a trusting relationship was key to getting patients to share what truly matters to them. The aim of asking ‘What matters to you?’ was not necessarily to fulfil all patient goals, but to get to know the patient. Research has demonstrated that patients want HCPs to take a holistic approach and know their preferences and personal circumstances, thereby fostering care continuity [58]. Aligning care with health priorities of older patients with multimorbidity improves patient outcomes and reduces unnecessary treatments [60]. The HCPs viewed continuity in assessments as crucial, allowing them to detect changes in patients’ fluctuating functional levels. Research has underscored long-term patient-HCP relationships’ role in nurturing relational care continuity for individuals with complex care needs [54]. Assessments can enhance relational continuity as HCPs use time to establish enduring therapeutic relationships with patients [26, 54]. Conversely, time constraints may hinder care continuity, along with the inability to frame assessment conversations appropriately [54].

In our study, the HCPs emphasised the importance of a multidisciplinary approach in forming comprehensive assessments from various professional perspectives, aligning with an umbrella review on integrated care for older patients with multimorbidity [61]. Furthermore, our findings align with the ICOPE framework [16], which highlights person-centred assessment in primary care as being essential for understanding patient preferences and identifying care and support needs. We found that assessment functions as a boundary object for internal and external interaction by providing a shared framework for discussing patient needs and coordinating care across disciplines. Boundary objects are referred to as means of collaboration [62, 63] between HCPs and services, and can facilitate boundary crossings. The HCPs viewed assessments as valuable objects upon patient transfers, e.g., from rehabilitation to home. However, the HCPs stressed the importance of shared goals and joint understanding and interpretation of assessment results to avoid fragmented services. Sharing assessment results and goals with collaborating HCPs was viewed as essential in facilitating continuity and counteracting fragmentation caused by different journal systems. Previous research has emphasised the importance of interprofessional communication [37, 56], shared patient assessments and team goals. One study similarly found that collaborating HCPs develop shared understandings of what makes care more person-centred through ‘knowledge bonding’ among HCPs [62]. Our findings emphasise that assessments used by HCPs within teams and upon patient transitions play a crucial role in fostering informational care continuity, aligning with previous research [26, 54, 64].

Participating municipalities have created their own assessment instrument, indicating a lack of a standardised instrument to accommodate their needs for consolidating multidisciplinary assessments into a shared form. Our findings align with research stating that the lack of a standardised instrument makes assessing frailty in patients with multimorbidity more challenging, also due to the lack of single frailty markers [53]. Although comprehensive geriatric assessment is viewed as the gold standard within hospital care for older patient with multimorbidity [65], it is not recommended for routine use in municipal healthcare because of resource constraints [25]. Our results point to HCPs’ importance in understanding the patient journey, which they felt relied on how information was transferred among care providers, as well as assessment inconsistencies. The HCPs were concerned that limited time and emphasis on quick discharges made it difficult to conduct thorough assessments and provide individualised care. Poor communication between services compounded these challenges further. Our results align with previous research highlighting collaboration within and across professional teams as being essential for achieving care continuity [54].

The HCPs faced increased coordination demands due to patients with complex needs. Previous research has emphasised vulnerabilities in health services [66] arising from compromised continuity due to resource constraints, communication gaps [37, 56] and care coordination challenges. The HCPs explained that inadequate assessments hindered identifying patients who require interdisciplinary services. Rigidity within the system prompted many HCPs to question their capacity to provide quality care, leaving them often feeling rushed and contributing to service discontinuity and duplicated efforts. The HCPs were frustrated with the system’s preference for efficiency over comprehensive assessments, raising concerns about compromised patient care quality. Previous research has identified challenges in operationalising care continuity [32], and a meta-ethnography highlighted tensions between balancing person-centred and efficient care [67]. The study underscored how prioritising efficiency potentially could hinder care quality if not balanced [67]. Another recent meta-ethnography [37] found that interprofessional communication is fundamental to consistent care, as patients want HCPs to talk to each other, which makes them feel safe [37]. Assessments have the potential to facilitate managerial continuity by linking patient journeys through better communication between HCPs, care consistency and holistic coordination [26, 54, 64, 68].

### Implications for practice and research

Our findings pose important implications for both practice and research. For practice, the findings could improve standardised assessments and collaboration among HCPs, and guide training programmes to enhance assessment skills and care continuity. As for research, the findings could generate further investigation into the challenges and optimal strategies for implementing comprehensive assessments across various healthcare settings.

## Methodological considerations

This study provides a contextual understanding of HCPs’ experiences and preferences in assessing multimorbidity among older adults in municipal healthcare settings. Our qualitative approach provided rich, detailed insights that emphasised HCPs’ voices and highlighted assessments’ holistic nature. The relatively small sample of HCPs may not have captured the full diversity of experiences and practices across settings. Furthermore, the sample comprised only women, which could limit any insights. More women than men work in this setting, but our results nevertheless represent a women’s perspective, which may differ from male HCPs. The participating HCPs came from diverse educational backgrounds, had varying experience levels, and worked in different units, offering detailed insights [44]. Additionally, healthcare leaders recruited HCPs for this study, which could have influenced the results if only the top HCPs were chosen, or others who might have contributed important perspectives were excluded. Potential pressure from leaders to participate was mitigated by offering participants the chance to contact the first author directly, as well as the option of withdrawing from the study at any time. The first author’s lack of research experience was balanced by an extensive clinical background, complemented by the second and third authors’ extensive research expertise. All authors actively participated in the analytical process, which enhanced our results’ trustworthiness by incorporating diverse perspectives and expertise. Rich descriptions of context and the participants allow the reader to assess our findings’ applicability [69]. Although our findings are specific to municipal healthcare and may not be easily generalisable to other contexts or healthcare systems, the findings may pose important implications for stakeholders and researchers interested in current topics. The study results were reported in accordance with Standards for Reporting Qualitative Research (see checklist in Additional file 3) [70].

## Conclusion

Based on interviews conducted with HCPs, our study highlights that comprehensive assessments go beyond standardised instruments. Assessments require a combination of clinical skills, structured instruments and unstructured methods, such as observations and informal conversations. These comprehensive approaches enable HCPs to foster trust, gain deep insights into patients’ lives, and promote relational continuity by understanding what truly matters to each patient. Furthermore, assessments are critical for facilitating interdisciplinary collaboration and ensuring informational continuity. Assessments serve as boundary objects that support interaction within internal teams and with external partners, ensuring that all stakeholders are aligned in their understanding and approach to patient care. Assessments can enhance managerial continuity by linking patient journeys and promoting coordination across services through better communication and shared understanding among the HCPs. While assessments can enhance care continuity, their effectiveness depends on several factors. The HCPs emphasised the need for sufficient time and professional expertise to conduct thorough assessments and facilitate interdisciplinary collaboration. However, linking patient journeys and seamless transitions between different care levels are hindered by fragmented services due to resource constraints and poor communication. Furthermore, inconsistencies in practices arising from insufficient training and lack of a standardised assessment instrument, along with disconnected journal systems and underutilisation of assessments, complicate care continuity further. Our results can benefit HCPs, policymakers and primary care providers by emphasising the need for a standardised assessment instrument, systematic and structured use of assessments, and a comprehensive approach to assessments to improve interdisciplinary collaboration and personalised care for older, home-dwelling individuals with multimorbidity.

## Supplementary Information

Supplementary Material 1.

Supplementary Material 2

Supplementary Material 3

## Data Availability

The data generated and analysed in this study are not publicly available due to terms of data collection approval. See Additional files 1 and 2 for interview guides.

## Abbreviations

HCPs: Healthcare professionals
FG: Focus group
ICOPE: Integrated Care for Older People
