# Supplementary material for: Assessment of older persons with multimorbidity in Norwegian primary care: a qualitative study of healthcare professionals’ experiences and preferences in fostering continuity of care
Source: BMC Health Serv Res. 2025 Jan 2;25:6. doi: 10.1186/s12913-024-12185-4 (PMC11694464; doi:10.1186/s12913-024-12185-4)
Supplement: Supplementary file 1 — Supplementary Material 1. [file 12913_2024_12185_MOESM1_ESM.docx]

Additional file 1

# **Interview guide – Focus group interview**

| **ENTRY QUESTIONS** |
| --- |
| 1. Can you please discuss what you do and how you work in the municipality with older people with multimorbidity?  - How do you understand multimorbidity? - How many people with multimorbidity do you meet? - How do you work with people with multimorbidity compared to other patients? |
| **ASSESSMENT METHODS AND EXPERIENCES** |
| 1. Can you please discuss what you do when assessing older people with multimorbidity?  - Which instruments do you use? - Who do you assess with these instruments? - Are the instruments useful (helpful)? How? Why not? |
| **SAFEGUARDING THE PATIENT’S VOICE** |
| 1. Can you please discuss how you safeguard the patients’ voices during assessments?  - Are there any related promoting or inhibiting factors? Which? - How do you assess what is important to patients? - How do you decide what assessments to make? |
| **BARRIERS AND FACILITATORS FOR ASSESSMENT** |
| 1. Can you please discuss what facilitates and what hampers assessments?  - Appropriateness of instruments? - Patient-related barriers? - What works, and why? |
| **CONCLUDING QUESTION** |
| 1. Are there other important issues you want to share that still needs to be mentioned? |
